# Prototyping Apps for the Management of Sleep, Fatigue, and Behavioral Health in Austere Far-Forward Environments: Development Study

**DOI:** 10.2196/40640

**Published:** 2023-08-28

**Authors:** Anne Germain, Megan Wolfson, I Wayan Pulantara, Meredith L Wallace, Katie Nugent, George Mesias, Kristina Clarke-Walper, Phillip J Quartana, Joshua Wilk

**Affiliations:** 1 Noctem, LLC Pittsburgh, PA United States; 2 Department of Psychiatry University of Pittsburgh School of Medicine Pittsburgh, PA United States; 3 Center for Military Psychiatry and Neurosciences Walter Reed Army Institute of Research Silver Spring, MD United States; 4 TechWerks, LLC Silver Spring, MD United States

**Keywords:** military digital health technology, operational environment, self-monitoring, self-management, connectivity protocol, evidence-based practice, deployment health, military, army, smartphone app, mHealth, mobile health, health app, feasibility, prototype, digital health, health technology, eHealth, decision support, medic, soldier, sleep, fatigue, behavioral health, operational setting, mental health, mental well-being

## Abstract

**Background:**

Military service inherently includes frequent periods of high-stress training, operational tempo, and sustained deployments to austere far-forward environments. These occupational requirements can contribute to acute and chronic sleep disruption, fatigue, and behavioral health challenges related to acute and chronic stress and disruption of team dynamics. To date, there is no centralized mobile health platform that supports self- and supervised detection, monitoring, and management of sleep and behavioral health issues in garrison and during and after deployments.

**Objective:**

The objective of this study was to adapt a clinical decision support platform for use outside clinical settings, in garrison, and during field exercises by medics and soldiers to monitor and manage sleep and behavioral health in operational settings.

**Methods:**

To adapt an existing clinical decision support digital health platform, we first gathered system, content, and context-related requirements for a sleep and behavioral health management system from experts. Sleep and behavioral health assessments were then adapted for prospective digital data capture. Evidence-based and operationally relevant educational and interventional modules were formatted for digital delivery. These modules addressed the management and mitigation of sleep, circadian challenges, fatigue, stress responses, and team communication. Connectivity protocols were adapted to accommodate the absence of cellular or Wi-Fi access in deployed settings. The resulting apps were then tested in garrison and during 2 separate field exercises.

**Results:**

Based on identified requirements, 2 Android smartphone apps were adapted for self-monitoring and management for soldiers (Soldier app) and team supervision and intervention by medics (Medic app). A total of 246 soldiers, including 28 medics, received training on how to use the apps. Both apps function as expected under conditions of limited connectivity during field exercises. Areas for future technology enhancement were also identified.

**Conclusions:**

We demonstrated the feasibility of adapting a clinical decision support platform into Android smartphone–based apps to collect, save, and synthesize sleep and behavioral health data, as well as share data using adaptive data transfer protocols when Wi-Fi or cellular data are unavailable. The AIRE (Autonomous Connectivity Independent System for Remote Environments) prototype offers a novel self-management and supervised tool to augment capabilities for prospective monitoring, detection, and intervention for emerging sleep, fatigue, and behavioral health issues that are common in military and nonmilitary high-tempo occupations (eg, submarines, long-haul flights, space stations, and oil rigs) where medical expertise is limited.

## Introduction

Military service is a demanding and high-risk occupation that includes frequent periods of accelerated operational tempo and deployments to austere far-forward environments. These occupational demands contribute to curtailed and disrupted sleep, circadian misalignment, fatigue, and a variety of behavioral health challenges including stress and compromised team dynamics. Together, these challenges degrade readiness and mission performance, impair critical cognitive abilities, increase the risk of medical and psychiatric disorders, and elevate the risk of injuries and costly or fatal mishaps [1,2]. For example, acute combat stress can incapacitate a soldier, or prolonged stress can lead to marked performance degradation, which may in turn require a medical evacuation, and adversely impact team functioning [3,4]. Over the past decade, psychiatric conditions have been among the most common reasons for medical evacuations [5-7].

The Department of Defense and military leadership have increasingly recognized the negative consequences of insufficient sleep and fatigue, behavioral health challenges, and poor team dynamics on armed forces readiness and performance [8-12]. In response, several new initiatives and programmatic changes have been implemented. For example, the Department of the Army renamed its physical readiness training field manual to *Health and Holistic Fitness* and updated it to include information on sleep management, mental readiness, relationships and communication, and mindfulness training [13]. The Air Force Integrated Resilience Directorate sponsored research for the Getting to Outcomes content area module for Air Force sleep health promotion to develop appropriate sleep promotion initiatives, and the US Navy has implemented scheduling changes that are more aligned with circadian rhythms [14-16]. Similarly, the Army’s Center for the Army Profession and Leadership added assessment tools and training focused on building mutual trust within a squad [17]. Although these changes are helpful, fewer initiatives have focused on increasing support for soldiers when they are in far-forward environments with less support and more austere conditions, and they face surges of personal and operational stress. In future combat environments, it is expected that behavioral health service resources will become even more limited [18], yielding greater demand on forward medical assets, like medics, to address sleep and behavioral health problems without specialty behavioral health support [9].

Digital technology can upskill medics and their capabilities to monitor and optimize sleep and behavioral health across a range of military-relevant settings, from those in garrison to far forward. Clinically focused mental health mobile apps are generally positively received by service members, and empirically driven, digital behavioral health interventions can support performance, safety, and health [19-21]. However, the nature of military occupational requirements in garrison and in austere far-forward environments (where sustained operations for prolonged periods of time, with minimal opportunity for sleep, behavioral health interventions, or personnel replacement, often occur) creates unique challenges for implementing mobile health–based tools [22]. Specifically, 4 factors limit the use of mobile health apps in military operational environments to support the detection, prevention, and management of sleep and behavioral health challenges. First, few existing mobile apps have capabilities to prospectively collect, analyze, and share data from multiple domains (ie, sleep, fatigue, behavioral health, team dynamics, and acute stress reactions) and capabilities to do so in a closed communication circuit, that is, without cellular or Wi-Fi network communication. Second, current apps do not support the concurrent monitoring of sleep, fatigue, behavioral health, and team dynamics at the team level by medical assets or leadership when such personnel become available. Third, few apps have the built-in capability for just-in-time and immediate assessment and management of acute and prolonged combat stress reactions. Finally, available apps provide no or limited evidence-based, context-relevant, and actionable recommendations for sustained mission readiness to the individual or to medical assets or leadership before, during, and after high-intensity training exercises or prolonged operations and deployments.

This study aimed at overcoming these challenges. Specifically, we adapted a validated clinical decision support tool designed for use by health care providers in clinical settings [23] into a field-deployable self-managed and supervised digital tool for use by soldiers and medics in the monitoring and management of sleep, fatigue, stress, and team dynamics in garrison and during military deployment in austere far-forward environments. Sleep, fatigue, stress, and team dynamics assessments, as well as related evidence-based educational and interventional materials, were adapted for this prototype. The secondary objective was to conduct a feasibility test of the resulting AIRE (Autonomous Connectivity Independent System for Remote Environments) prototype, consisting of a Soldier app and a Medic app, in active-duty service members who were engaged in field exercises that simulated austere far-forward environments. Here, we summarize how system requirements were identified and operationalized for the AIRE prototype, specific features and functions of the resulting Soldier and Medic apps, and opportunities for further enhancement.

## Methods

### Requirement Analysis

A requirement analysis was conducted based on content and contexts of monitoring and management through a digital platform for use by military personnel during day-to-day operations in garrison and military field training exercises or deployments. Sleep and fatigue context and contents focused on prospective daily assessments (daily check-ins), educational information regarding healthy sleep practices and factors that promote or disrupt sleep, and evidence-based behavioral techniques to support sleep optimization and fatigue mitigation. Stress and team dynamics context and contents focused on daily assessments and recurrent measurement of key components of perceived team dynamics, educational information regarding stress and stress management, optimal communication techniques, and evidence-based methods to mitigate stress and interpersonal conflicts. For stress and team dynamics, context and content requirements were gathered from a previous qualitative study with soldiers who had been deployed as part of small teams in austere isolated environments. Soldiers emphasized that common problems experienced in these environments included impaired functioning from operational and personal stress and disruptions in team communication. Additionally, medics reported receiving little prior behavioral health training, and many said that they did not feel comfortable addressing behavioral health concerns with their soldiers [24].

For educational and interventional materials, a literature review guided the identification of evidence-based, safe, and effective sleep and fatigue mitigation strategies, behavioral health optimization strategies, and conflict mitigation strategies in military deployment settings or similar high-risk, high-tempo occupations. Relevant directives and instructions regarding sleep and fatigue management from the Department of Defense were also reviewed to extract specific requirements and verify alignment between instructional content and published recommendations [13,25,26]. For behavioral health and team dynamics, including conflict mitigation, previous and current Department of Defense trainings and supporting scientific literature focused on brief interventions were reviewed.

Functional requirements included assessment data collection, security and user authentication, role-based access based on user types (ie, medic or soldier) and their team, anonymity of aggregate data, reminders and notifications, accessibility of team-level aggregated data by medics, time-based summary statistics to soldiers, and capability for 1-way secure SMS text messaging from medics to their soldiers. System-wide requirements included Android-based devices and operation system; activation of GPS location limited to active data transfer; adaptive data transfer and synching protocols when Wi-Fi or cellular data were not available, such as Bluetooth Low Energy (BLE); and activation of GPS location, which is required to enable offline data transfer in Android devices. Additional nonfunctional requirements included the ease of access and distribution of the apps, safeguards for assurance and reliability of the overall system, assurance of security and privacy of data at rest as well as in transit, a method for maintaining a separation of concerns, and development of a recovery procedure in the event of service disruption to minimize downtime.

### Key Opinion Leader Interviews and Additional System Requirements

To augment and advance requirements detailed above and collected from previous studies [24], we used a participatory design approach with representative members of the intended user base (ie, soldiers and military medical personnel). Specifically, we built on initial findings and used semiqualitative interviews to focus on functional requirements that would optimize usability and impact in far-forward environments. All interviews were conducted remotely because of the COVID-19 pandemic. Interactive mock-ups were presented to interviewees to depict the overall architecture of the anticipated prototype, workflows, and user interactions with the apps, data display, and content presentation. After each interview, suggested modifications were iteratively integrated into a new set of mock-ups that was reevaluated in the following interview. Qualitative semistructured interviews that were completed with 6 key opinion leaders (KOLs) in military health with deployment experience augmented the requirement analysis to obtain additional information on functional and nonfunctional requirements needed for maximal applicability and utility in austere far-forward environments. Interviews were completed between May 8, 2020, and June 10, 2020, by a coauthor (MW) who has extensive experience in quantitative and qualitative research methods in military populations.

Interviewees first received a description of the purpose of the interview and how the information gathered would be used to develop a military-relevant, context-sensitive digital platform for the prospective monitoring and management of sleep and behavioral health issues. The semistructured interview focused on environmental and habitability-related factors; personal, social, and mission-related factors that impact sleep and behavioral health across military operational contexts; and perceived facilitators and barriers to self- and supervised monitoring and management of sleep and behavioral health in these contexts. Participants were presented with medium-resolution interactive mock-ups of the Soldier and Medic AIRE apps and were asked to comment on the ease or complexity of the user workflow to evaluate different approaches for the selection and graphic display of information collected from self-report sleep and behavioral health metrics. They also were asked to provide feedback on the planned assessments for relevance and feasibility and to maximize user completion. In the process, participants were asked to identify key (“must-have”) requirements to optimize utility of the envisioned platform versus other, “nice-to-have” requirements.

### Designing the Soldier and Medic Apps

**Figure 1:**
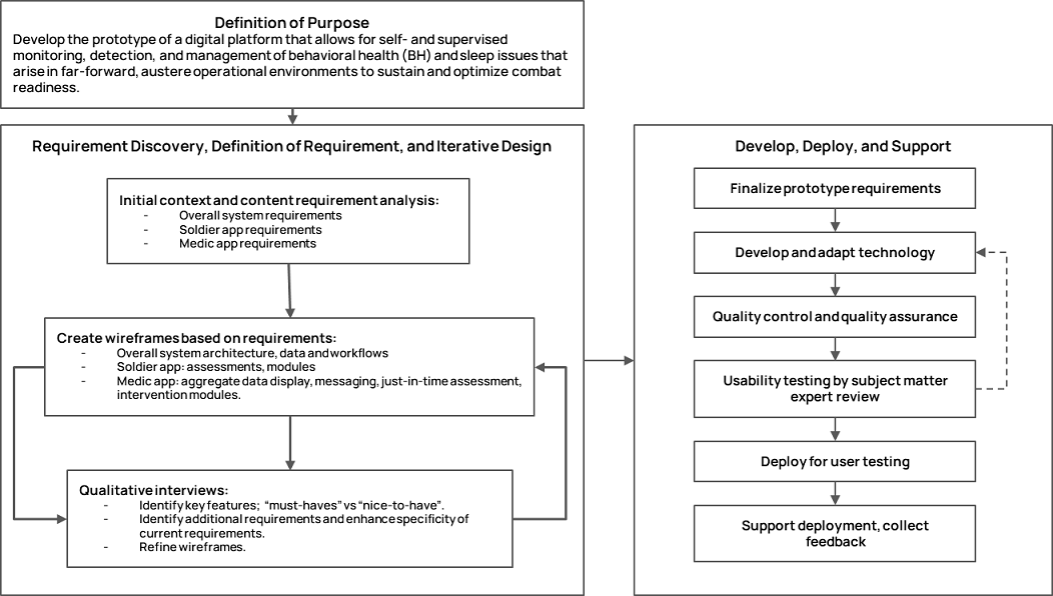
Summary of the iterative process for requirement gathering and app design.

Figure 1 summarizes the prototype design process.

We used the user-centered design process [27] in designing the Soldier and Medic mobile apps. As such, designs were based on an explicit understanding of users, tasks, and their environments, which are the direct results of the requirement analysis described above. Because the users’ roles and tasks are different between the Medic and Soldier apps, the design emphasis of each app was adjusted accordingly. The emphasis on the Soldier app’s design was to provide an efficient user interface and experience (UI/UX) in answering assessment questions (eg, daily check-ins) while also providing easy-to-follow UI/UX for the recommended modules. The Medic app focused on medics’ requirements to understand the current status of soldiers under their care, a streamlined process to suggest relevant modules to send to soldiers based on their status, and the ability to monitor review of the recommended modules.

Medium-fidelity wireframes of each page of the Soldier and Medic apps were created to outline the types, layout, and organization of information on each app page. These wireframes were iteratively refined until congruence between initial requirements and design was achieved. High-fidelity prototypes included detailed visual design and styling. Because of restrictions in accessing non–government-secured web-based user-centered design tools typically used to quantify user behavior, detailed transcriptions of the feedback and information gathered during the qualitative interviews were reviewed by the team to extract technical and functional requirements and inform the revisions of the interactive wireframes presented at subsequent interviews. These prototypes underwent usability testing by 2 KOLs for feedback on UI/UX elements and interactions.

### Recruitment

This study, titled Adapt and Improve in Remote Environments, was command-directed. Choosing a command-directed study enabled the research team to engage with and address service members of an entire brigade for participation in the study. Participants were soldiers and medics from 2 brigade combat teams who were scheduled for field training exercises. A total of 246 soldiers and medics (218 soldiers and 28 medics) completed the in-person AIRE orientation training and received a study-issued smartphone to access and use the Soldier and Medic apps.

At the orientation training, a nonmilitary member of the study team provided an overview of the study, including a description of measures to protect confidentiality. All participants were educated about their rights as research participants and made aware of their ability to discontinue participation at any time without any consequences. Soldiers and medics were asked to volunteer for the research portion of the study via the baseline survey and were given a participant information sheet to keep for their records. Participants were asked to indicate on the electronic survey whether they agreed to allow their responses on the apps to be used for research purposes.

### Ethics Approval

The Walter Reed Army Institute of Research institutional review board approved the study (20910-7500).

### Soldier and Medic Apps Training

Soldiers and medics received a smartphone on which the respective Soldier app only or the Soldier and Medic AIRE apps were installed. For both soldiers and medics, the orientation training detailed how to identify sleep, fatigue, and behavioral health problems; self-management intervention strategies; and how the AIRE app could assist in managing sleep, fatigue, behavioral health, and team dynamics problems. Practical knowledge and direct training on the use of the app were provided. Medics received additional education and training about how to monitor soldier use of the app and recommend intervention modules when appropriate.

### App Use in Garrison

To gain proficiency in using the AIRE apps, the soldiers and medics were instructed to use the app daily for approximately 2 weeks in garrison before their field training exercise. During this time, they were able to contact the study team regarding questions and concerns. In addition, the AIRE apps included a “tech support” feature that was enabled when Wi-Fi connectivity was available. This feature allowed users to anonymously send questions and concerns to the study team.

### App Use in the Field Training Environment

The units left their respective military bases for field training in summer and fall of 2021. While at the field training, soldiers and medics were instructed to use the Soldier app to complete daily assessments and review recommended educational or interventional modules. Modules were either recommended directly to soldiers by the Soldier app or sent by their medic based on either the medic’s clinical determination or recommendations by the Medic app. While in the field, all data were collected, recorded, and transferred between the Soldier and Medic apps via BLE protocols.

### Post–Field Test Procedures

Upon return to garrison, soldiers and medics completed an assessment of the usability of the Soldier and Medic apps (data not reported here). Smartphones were returned, and final device synching to a cloud upload was completed.

## Results

### Requirements

**Figure 2:**
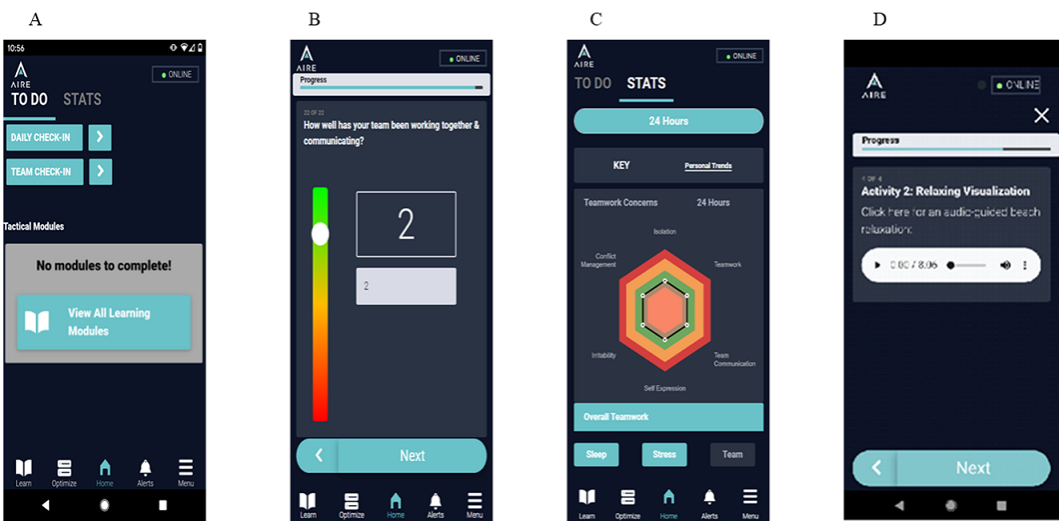
Soldier app. (A) Soldier app dashboard. The to-do list instructs soldiers to complete available assessments and modules highlighted in teal. Gray items indicate completed items and time remaining until assessment needs to be completed again. In this example, the recommended modules to complete are based on self-report assessment. (B) Example of item presentation from the sleep portion of the daily check-in. (C) Example of summary data from soldiers’ self-report for team dynamics that depicts self-report for the past 24-hour period. Summaries for the sleep or stress domains are displayed by pressing their respective related buttons. (D) Example of a self-management module for guided relaxation with audio content.

**Figure 3:**
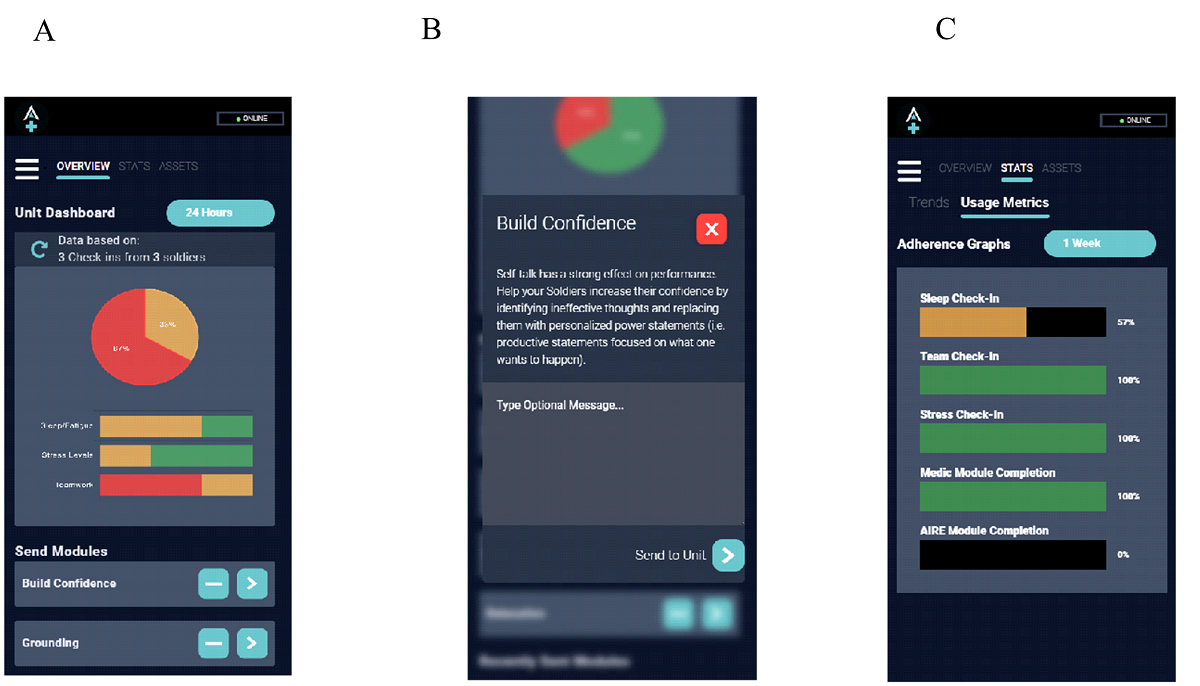
Medic app. (A) Medic app dashboard presents the summary of aggregated data from assigned soldiers who completed assessments for a preselected period (herein, 24 hours). On the basis of aggregate soldiers’ responses, medics receive recommendations for modules that can be sent to their soldiers’ app. (B) Medics can send selected modules to their group of soldiers with or without the addition of a text message. (C) Medics can monitor the rate of completions of check-ins and self- or medic-recommended modules for their assigned soldiers.

From interviews with KOLs, 4 common requirements emerged to optimize the usability of a military-relevant, deployable, self-management and medic-monitored digital health platform for use in far-forward environments. First, the apps must be adaptive to resources available in field environments and mission-related demands, must be responsive, and must consider the intermittent availability of a power supply to recharge devices. Thus, we limited the need for frequent device recharge by keeping the apps’ power use minimal by optimizing the network usage, by minimizing the app footprint to under 75 MB, by using BLE instead of the energy-intensive Bluetooth class 2 normally used in mobile apps, and by optimizing the BLE broadcasting or advertising frequency. Users also received solar chargers to charge smartphones during field exercises. Second, information and recommendations must be clear, brief, and actionable. To meet this requirement, all assessments and educational and interventional materials were designed to support the succinct and user-friendly display of information, including clearly labeled custom graphic diagrams, short text files, and audio files when appropriate. For repeated assessments, short measures of sleep, fatigue, stress, and team dynamics were adapted from validated items and scales as needed. Sleep and stress assessments consisted of a daily self-report check-in including 18 items that required less than 3 minutes to complete, and team dynamics check-ins consisting of 6 items that required less than 1 minute to complete. Third, the apps must tap into soldiers’ competitive nature. To meet this requirement, we used a green, amber, or red color scheme to graphically display self-report scores on the Soldier app and the number of soldiers with scores in each category for the Medic app. On the Soldier and Medic apps, green indicated that scores were within expected healthy ranges, amber indicated that scores were suboptimal, and red indicated scores in the critical range. For example, on the Soldier app, self-reported sleep duration was in the green category for sleep ≥7 hours, amber for 5 to 7 hours of sleep, and red if a soldier reported less than 5 hours of sleep (Figure 2). On the Medic app, the same color scheme represented the percent of soldiers whose scores were within normal or healthy parameters (green), below the healthy range but above operationally problematic range (amber), and in the operationally problematic range (red); for example, green corresponded to percent of soldiers sleeping ≥7 hours. Our usability testing with KOLs supported that this color scheme (green, amber, or red) was recognizable and meaningful among soldiers and medics. Lastly, the Medic app must provide team-level metrics to the leadership and medics to make team-relevant recommendations and decisions. To address this requirement, the Medic app displayed team-level aggregate data only. Individual soldier data were not presented on the Medic app. As displayed in Figure 3, the landing page of the Medic app was designed as an easily accessible, at-a-glance team status overview.

A sample of key features created based on requirements for the Soldier and Medic apps are described in Table 1. Additional information is provided in the presentation of the Soldier and Medic apps in the next section.

**Table 1:** Examples and description of features of the Soldier and Medic apps.

Feature	Description	App
Brief assessments with input validation	Repeated assessments designed for completion within minutes. Ensure that each entry field is within the correct data range and warn users if it is out of bounds. Invalid or missed entries are highlighted for correction by user (eg, incomplete items).	Soldier
Longitudinal self-report data display	Display changes and trends in an individual’s behavioral health data per domain and overall.	Soldier
Actionable recommendations	Brief modules with specific and interactive steps for how to implement the technique.	Soldier
Notifications and reminders	Soldier receives notifications when their medic sends a message, an assessment, or new information for completion or review. Soldier receives reminders for scheduled self-report assessments.	Soldier
Self-help resources	Brief educational material and intervention suggestions for managing problems with stress, sleep, and team communication problems that are available to everyone (ie, not tied to an assessment score).	Soldier
Aggregate data display	Aggregate summaries of the number of soldiers’ scoring in the predetermined score range for self-report measures over a selected period.	Medic
Adherence monitoring	Aggregate summaries of soldiers’ engagement with app-based assessment and modules.	Medic
Secure unidirectional messaging system	Medics can send a message to their unit to share reminders, information, or assessments.	Medic
Behavioral health helper resources	Several modules to assist medics with communicating with their soldiers about behavioral health concerns; identifying stress, sleep, and team functioning problems; and assisting with management of identified problems.	Medic
Time-sensitive assessment of acute stress reactions	Medic can send an assessment to evaluate acute stress reactions at any time.	Medic and Soldier

### Soldier and Medic Apps

The AIRE platform prototype includes Android-based Soldier and Medic apps. Both apps function independently and as a system irrespective of a connection to a cellular network or Wi-Fi. The Soldier app (Figure 2) is used by soldiers to input self-report assessments of sleep, fatigue, and stress symptoms (daily check-in) and team dynamics (every 72 hours). In addition, medics could elect to send a just-in-time assessment of acute stress reactions in the event of a critical incident. A customizable check-in reminder system was also implemented to support regular completion of the daily check-in at the same time each day. For each self-report assessment, validation checks were implemented to ensure data quality. This function supports immediate verification of responses and directs the user to correct any invalid entries. For each soldier, the app graphically displayed self-report data for predetermined time frames of the past 24 hours, past 72 hours, past 7 days, and all data.

Self-management modules were automatically recommended to the soldier on their app when their response on the daily check-in or team dynamics check-in exceeded predefined thresholds. All modules were based on evidence-based tactics to optimize sleep, manage fatigue and stress, and promote effective communication. Educational and self-management modules covered topics such as healthy sleep practices, impact of fatigue, grounding, visualization, tactical breathing, steps for conflict resolution, and optimizing one’s sleep environment. An icon indicated to the soldier that a module was recommended by the Soldier app, which differed from the icon when a module was recommended by their medic and sent to the Soldier app.

The Medic app (Figure 3) was designed to allow medics to monitor their soldiers’ self-report assessments of sleep, fatigue, stress, and team dynamics in aggregate. The Medic app also allowed medics to monitor the rates of completion of assessments and recommended modules by their soldiers. In addition, medics received recommendations for modules to offer to their soldiers based on the aggregate soldiers’ self-report assessments, which they could either accept or decline. As identified in KOL interviews, the landing page of the Medic app provided an easy-to-interpret overview of the status of their assigned soldiers globally across all 3 measured domains (pie chart) as well as for each individual domain of sleep and fatigue, stress, and group dynamics.

To further assess team-level aggregate trends, medics were able to access and review group-level assessment metrics for each domain of interest. Consistent with the Soldier app, these metrics were displayed for the time frames of the past 24 hours, past 72 hours, past 7 days, and all data.

When the proportion of soldiers’ responses on an assessment exceeded predefined thresholds, related education and intervention modules automatically appeared on the dashboard of the Medic app as a recommendation to mitigate potential soldier-reported difficulties. To protect soldiers’ confidentiality, data were only displayed to the medic when more than 1 soldier’s responses were available on a given assessment. Medics determined whether to send the recommended modules to their soldiers. For each module forwarded to soldiers, the medic could also include an SMS text message with clarifying instructions. As stated above, medic-recommended modules were clearly identified on the Soldier app with a unique icon. Medics could recommend any modules included on the Soldier app. In addition, medic-specific modules included basic steps for imagery rehearsal therapy for nightmares, sleep banking, instructions to mitigate jet lag, and tactics for managing intrusive thoughts before sleep. All information, strategies, and best practices reviewed with medics during the medic training were also available on the Medic app as an additional resource or “tool kit” for them to reference. Finally, medics were able to monitor soldier adherence to completing self-report assessments and which recommended modules were completed. The same color scheme represented adherence to scheduled and recommended modules (eg, ≤50% displayed as red, 50%-70% as amber, and ≥70% as green).

### Participants

A total of 246 soldiers, including 28 medics, received a study-issued smartphone that included the Soldier app. Medics’ smartphones also included the Medic app. A total of 28 (11.4%) of the issued smartphones were damaged or not returned, did not have data, or had inaccessible data upon return of the devices. Baseline assessment data were missing for 84 soldiers and 11 medics; this was due to either soldiers’ improper submission of the assessment on the app or issues with the completed assessment uploading to the cloud when a connection was available. Of those soldiers who completed the baseline assessment, 125 nonmedic soldiers (93% consent rate) and 16 medics (94% consent rate) gave consent to participate in the study. Sample characteristics are summarized in Table 2. A detailed report of the feasibility study results is published elsewhere (Nugent et al, manuscript under review).

**Table 2:** Participant characteristics.

Characteristics		Soldiers (n=125), n (%)	Medics (n=16), n (%)
Male gender		108 (86.4)	14 (88)
**Age (years)**			
	18-21	42 (33.6)	5 (31)
	22-25	34 (27.2)	6 (38)
	26-29	25 (20)	3 (19)
	30-41	24 (19.2)	2 (13)
**Race**			
	White	84 (67.2)	11 (69)
	Black or African American	13 (10.4)	0 (0)
	Asian	6 (4.8)	3 (15)
	Native Hawaiian or Pacific Islander	6 (4.8)	0 (0)
	American Indian or Alaska Native	2 (1.6)	0 (0)
	Multiracial	14 (11.2)	2 (13)
Hispanic or Latino ethnicity		18 (14.4)	3 (19)
**Education**			
	High school or General Education Development	67 (53.7)	4 (25)
	Some college or associates	44 (35.2)	10 (63)
	Bachelor’s degree	12 (9.6)	2 (13)
	Graduate degree	2 (1.6)	0 (0)
**Rank**			
	Junior enlisted	80 (63.6)	11 (69)
	Senior enlisted	38 (30.4)	5 (33)
	Lieutenant/captain	7 (5.6)	0 (0)
**Marital status**			
	Single, never married	62 (49.6)	5 (31)
	Married	54 (43.2)	11 (69)
	Separated or divorced	8 (6.4)	0 (0)
Received help for a behavioral health or sleep problem past year		19 (15.2)	6 (38)^a^
Previously used a health-related app^b^		21 (60)	6 (100)

^a^Significant difference between groups; *χ*^2^_1_=5.42, *P*=.02.

^b^Data available for a subsample of 35 nonmedics and 6 medics.

In total, 1561 self-report daily check-ins, team check-ins, and stress check-ins were exportable from the 1603 records created via the Soldier app in garrison and during the field exercises from soldiers who provided consent for data use. Less than 3% of data exportable records were corrupted or missing. Failed synchronization of locally saved data to a cloud-based database and incomplete assessments accounted for data loss.

The most frequently reported technical difficulties reported by medics and soldiers included slow data transfer due to intermittent connectivity between the Medic and Soldier apps while in garrison or during the field exercise (n=9) and challenges with charging the device during field exercises (n=7).

## Discussion

### Principal Findings

This study aimed at adapting a clinical decision support platform designed for the management of insomnia and other behavioral sleep disorders in clinical settings [23] into a field-deployable smartphone-based platform designed to capture prospective self-report measures from soldiers and monitored by medics. The newly adapted AIRE platform provides a novel tool to assess, monitor, and manage sleep, fatigue, stress responses, and team dynamics in military operational settings efficiently and prospectively. More broadly, this platform offers an application architecture that can be used to assess, monitor, and manage performance domains that can be leveraged across multiple occupational settings in support of human performance optimization. We demonstrated the feasibility of collecting data and providing self-management techniques and medic-recommended strategies designed for the optimization of sleep, performance impacts due to stress, and group dynamics in garrison and during field exercises.

We first identified platform requirements with the input of KOLs with experience and expertise in military operations and military field research. Four primary functional requirements were identified to optimize the relevance and usability of the Soldier and Medic apps in far-forward environments, which directly informed the initial and final user interfaces for the Soldier and Medic apps. To capture the distinct purpose of each app, designs were iteratively refined with the input of KOLs to optimize timely data capture from soldiers and facilitate the interpretation of data and selection of appropriate recommendations by the medics. Soldiers’ and medics’ ratings of the app regarding the ease of use, understanding, and integration of functions suggest that the requirements were met and that the UI/UX design met the core requirements.

We demonstrated the feasibility of implementing the key requirement regarding the ability to use smartphone-based apps to record, save, transfer, and synthesize data using BLE when Wi-Fi or cellular data are unreliable or unavailable. This is relevant both in garrison—where Wi-Fi and cellular connectivity can be spotty—and for use in far-forward environments, including field exercises, where Wi-Fi and cellular data are unavailable. For this prototype, we selected to overcome connectivity issues and support secure data transfer from and to the Soldier and Medic apps. However, offline transfer rate is significantly slower in comparison with Wi-Fi or cellular data transfer. In an attempt to alleviate this issue, we focused on optimizing the transfer process to use as much BLE transfer bandwidth as possible while maintaining the reliability of the transfer process. Data loss was mainly due to failed synchronization of locally saved data to the cloud-based database, and the slower BLE transfer rate may have contributed to medic-reported issues with device connectivity. In future iterations of the AIRE platform, we will improve our BLE transfer algorithm and explore other secure data transfer technologies.

Overall, the resulting AIRE platform can readily be adapted for the monitoring and management of sleep, fatigue, stress, group dynamics, and other relevant functional domains of performance in other austere environments characterized by high tempo where connectivity and medical support are limited (eg, maritime transportation, long-distance hauling, and oil rigs).

### Limitations and Future Developments

Because this study focused on testing the feasibility of deploying newly adapted Soldier and Medic apps in garrison and during field exercises, the overall impact of the self-management modules or medic-recommended modules on different domains of performance was not evaluated. A randomized controlled trial will be required to evaluate the extent to which digital self-management tools such as AIRE may sustain or improve sleep, fatigue, stress, behavioral health, and team dynamics in garrison and during field exercises or during prolonged military deployments. A randomized clinical trial will also be necessary to evaluate the extent to which supervised monitoring and management by medics or behavioral health officers may enhance the impact of soldiers’ self-monitoring and management.

Finally, specific challenges unique to military deployments may not have been captured under the conditions of the field test trials conducted in this study.

Although we designed AIRE to readily allow for the integration of data collected from wearable or nearable devices, open application programmable interfaces of wearable and nearable vendors require a Wi-Fi or cellular connection for data transfer. Additional development will be required to advance BLE-supported data transfer protocols for the integration of wearables or nearables.

For the purpose of this study, only team-level aggregate data were displayed on the Medic app and only 1-way communication from medics to soldiers via the SMS text messaging system was enabled. However, capabilities are built into the platform for individual-level information and intervention, as well as a 2-way messaging system between soldiers and their assigned medic for future refinements and applications. Of note, individual and aggregate data can be impactful tools for use in garrison for behavioral health care providers and leadership and could ultimately augment readiness assessment and risk management tools, such as the Commander’s Risk Reduction Toolkit.

In summary, we have demonstrated the feasibility of a deployable digital monitoring and self-management and medic-supervised platform to monitor and optimize sleep, stress, and team dynamics in garrison and in a forward environment. In garrison and during field deployments, the AIRE prototype provides an example of technology that is relevant for service members and has the potential to augment resources for sleep and behavioral health management, as well as support enhanced critical aspects of team cohesion. For occupations in austere, far-forward settings with prolonged workdays (eg, submarines, long-haul flights, space stations, and oil rigs), capabilities for prospective monitoring and just-in-time personalized interventions in multiple areas of performance can provide critical support when medical expertise is not readily available or accessible.

## Abbreviations

AIRE: Autonomous Connectivity Independent System for Remote Environments
BLE: Bluetooth Low Energy
KOL: key opinion leader
UI/UX: user interface and experience
